# The University of Buffalo, Medical Department, Alumni Association Commencement

**Published:** 1889-04

**Authors:** 


### *THE UNIVERSITY OF BUFFALO, MEDICAL DEPARTMENT, ALUMNI ASSOCIATION COMMENCEMENT.*

The fourteenth annual meeting of the Alumni Association of the Medical Department of the University of Buffalo was held at the college building on the Commencement Day, March 26, 1889.

The Association convened in the upper amphitheater in the morning session, when Professor E. V. Stoddard, of Rochester, delivered the address of welcome. The President's annual address was deferred until the afternoon session. At the business meeting, the following officers were elected:

*President*, E. L. Shurley, Detroit; *First Vice-President*, Peter M. Wise, Ovid; *Second Vice-President*, Eugene Smith, Detroit; *Third Vice-President*, E. R. Hopkins, Silver Creek; *Fourth Vice-President*, M. B. Folwell, Buffalo; *Fifth Vice-President*, Alex. McNamara, Corning; *Permanent Secretary*, John J. Walsh, Buffalo; *Recording Secretary*, Eugene A. Smith, Buffalo; *Treasurer*, E. C. W. O'Brien, Buffalo; *Trustees:* D. W. Harrington, E. C. W. O'Brien, M. D., Buffalo, N. Y.; Horace Hoyt, M. D., East Aurora, N. Y.; Henry Lapp, M. D., Clarence, N. Y.; F. E. L. Brecht, M. D., Buffalo, N. Y.; *Executive Committee:* F. W. Abbott, H. H. Bingham, and W. T. Tanner.

Dr. W. Scott Renner, of Buffalo, and Dr. John McPherson, of Akron, N. Y., were elected honorary members.

The afternoon session was held in the lower amphitheater, when the Vice-President, Dr. E. L. Shurley, read his annual address, after which the following papers were read: "Practical Observations on the Localization of Diseases of the Brain and Spinal Cord," Frederick Peterson, M. D., New York; "Dilatation of the Heart and its Treatment," Henry R. Hopkins, M. D., Buffalo, N. Y.; "Rachitis," Irving M. Snow, M. D, Buffalo, N. Y.

Dr. Richmond, of Livonia, presented for the Association's consideration an interesting case of innominate aneurism.

The commencement exercises in the evening were held in Music Hall, where a large audience assembled to witness them. The degree of Doctor of Medicine was conferred upon the following candidates by the vice-chancellor, the Hon. James O. Putnam:

Albon E. Bartoo, Eden, N. Y.; Alfred Joseph Rutherford, Milwaukee, Wis.; Robert S. Carr, East Bloomfield, N. Y.; Anna Wadsworth, Brockport, N. Y.; William J. Zopfi, Buffalo, N. Y.; James H. McCort, Rochester, N. Y.; Jacob M. Kraus, Clarence, N. Y.; Grover W. Wende, Mill Grove, N. Y.; Leonard O. Eastman, Berkshire, N. Y.; Frederick W. Koehler, Buffalo, N. Y.; William Stanton, Belfast, N. Y.; Marrian A. Townley, Ludlowville, N. Y.; Patterson J. McNamara, Corning, N. Y.; Henry Jones Mulford, Buffalo, N. Y.; Augustus L. Damon, Canaseraga, N. Y.; Robert W. Green, Geneseo, N. Y.; Lewis E. Dixson, Morris, N. Y.; George W. Goler, Rochester, N. Y.; Lorenzo P. McCray, Clymer, N. Y.; Herbert A. Birdsall, Buffalo, N. Y.; Louis A. Ball, Buffalo, N. Y.; Allen A. Jones, Buffalo, N. Y.; John Theren James, Medina, N. Y.; Lyman C. Broughton, Buffalo, N. Y.; Edward Whittier Kendall, Kendall, N. Y.; Alfred W. Henckell, Rochester, N. Y.; Herbert Upham Williams, Buffalo, N. Y.; John M. O'Brien, New York City; John R. Gray, Buffalo, N. Y.; Elmer E. Briggs, Silver Creek, N. Y.; Arnold Drexel, Mount Calvary, Wis.; Samuel Huston Lynde, Buffalo, N. Y.; Ammiel J. Thorne, Haverlack, Canada; Lucius L. Ball, Buffalo, N. Y.; John C. Brown, Smithport, Pa.; Harold A. Hayes, Tonawanda, N. Y.; Katharyn M. Bailey, Buffalo, N. Y.; Walter S. Yates, Santa Barbara, Cal.; William T. Twitty, Warrenton, N. C.; Thomas Bagley, Buffalo, N. Y.; William O. Burbank, Gaines, N. Y.; Cornelius Mackey, Rushville, N. Y.; John J. Drake, Buffalo, N. Y.; William A. Ladd, Clarence, N. Y.; Thomas B. Fernald, Norwich, N. Y.; Walter E. Gregory, Dansville, N. Y.

The degree of Graduate in Pharmacy was conferred upon sixteen candidates.

These gentlemen all appeared before the Board of Twenty-two Curators in the morning, and were examined in obstetrics and gynecology, practice of medicine and surgery.

The address to the Alumni was delivered by Professor George Henry Fox, M. D., of New York, and the address to the graduating class was given by Ansley Wilcox, Esq., of this city.

An appropriate banquet at the Tifft House closed, in a most agreeable manner, a memorable day in the history of the College.

